# Cardiorespiratory fitness and its association with adiposity indices in young adults

**DOI:** 10.12669/pjms.333.12294

**Published:** 2017

**Authors:** Mozaffer Rahim Hingorjo, Sitwat Zehra, Zainab Hasan, Masood Anwar Qureshi

**Affiliations:** 1 Prof. Mozaffer Rahim Hingorjo, FCPS. Department of Physiology, Jinnah Medical & Dental College, Karachi, Pakistan; 2 Dr. Sitwat Zehra, PhD. Department of Physiology, Dr. A.Q. Khan Institute of Biotechnology & Genetic Engineering (KIBGE), Karachi, Pakistan; 3 Dr. Zainab Hasan, MSc. Department of Community Medicine, Jinnah Medical & Dental College, Karachi, Pakistan; 4 Prof. Masood Anwar Qureshi, PhD. Department of Physiology, Dow University of Health Sciences, Karachi, Pakistan

**Keywords:** Adiposity, Cardiorespiratory fitness, Exercise, Health education

## Abstract

**Objective:**

This study investigated the relationship between cardiorespiratory fitness (CRF) and adiposity in young adults.

**Methods:**

Data was collected from 133 students of a medical college of Pakistan. The study was conducted on young adults, aged 17-24 years, recruited from Jinnah Medical & Dental College, Karachi, between Aug-Dec, 2015. Queen’s College Step Test was conducted to measure CRF and maximal oxygen uptake (VO_2max_) evaluated. Anthropometric measurements (body mass index, body fat, visceral fat, waist circumference) were taken to assess adiposity. Associations of VO_2max_ and adiposity were analyzed.

**Results:**

The prevalence of overweight/obesity was 44% overall. The VO_2max_ (ml/kg/min) of males and females was 55.41±9.45 and 39.91±3.14, respectively, the gender difference being highly significant (*p*<0.001). Quartiles of VO_2max_ showed strong inverse relationship between adiposity and VO_2max,_ obese individuals having low VO_2max_ (1^st^ quartile) and normal weight individuals having high VO_2max_ (4^th^ quartile). VO2max correlated greatest with body fat in males (r = –0.600; p<0.001), and waist circumference in females (r = –0.319; p=0.004).

**Conclusion:**

The results indicate low CRF in young females and a strong inverse relationship between fitness levels and adiposity in young adults of both genders. Improving these parameters in our young population may prevent development of chronic non-communicable disease in later life.

## INTRODUCTION

Cardiorespiratory fitness (CRF) also referred to as *aerobic capacity* is one of the most important components of physical fitness. It is the ability of the body to perform dynamic, large-muscle exercise, for prolonged periods, at moderate-to-high intensity.^1^ Maximum oxygen consumption (VO2max) is considered to be the most widely accepted measure of CRF giving a baseline estimate of one’s heart and lung capacity and can be used to follow the progress of daily physical exercise.^2^

Studies have shown that high levels of cardiorespiratory fitness in young adults is associated with a lower risk of having calcification in the coronary arteries and prevents the development of early atherosclerotic vascular disease.^3^ Physical inactivity and sedentary behavior leads to accumulation of excess adipose tissue and a state of chronic inflammation which is a major factor in the development of non-communicable diseases (NCD). Data from the Pakistan Demographic and Health Survey 2013 shows that approximately 15% of the youth bulge in Pakistan is between the ages of 18 and 25 years, comprising mostly of college and university students. The physical activity levels in this age group are not enough to prevent NCD in later life.^4^ Regular physical exercise increases CRF levels and suppresses the chronic inflammatory state of obesity, lowering the risk for NCD.^5^

Measurement of VO_2max_ is therefore considered an important part in the evaluation of the cardiorespiratory health and aerobic fitness. Sophisticated laboratory equipment is required to measure aerobic capacity directly, however, it can reliably be estimated indirectly by performing submaximal exercise protocol such as the three minute Step Test.

Despite the importance of CRF and its role in prevention of NCDs in later life, there is lack of studies performed locally. Data on adiposity in young adults is also lacking. Additionally, it is important to analyze data on CRF and adiposity measures using gender disaggregation as there are differences in disease risk and implication. This paper provides a baseline evaluation of CRF levels, adiposity and their gender based correlations.

## METHODS

This was a cross-sectional study conducted on young adults, aged 17-24 years, recruited from Jinnah Medical & Dental College, Karachi, between Aug-Dec, 2015. All subjects completed Physical Activity Readiness Questionnaire, a simple questionnaire designed to make sure that there are no obvious reasons that a person should see a medical doctor before becoming much more physically active.^6^ Written informed consent was taken from each subject and the study was approved by the research and ethical committee of Jinnah Medical & Dental College, Karachi, Pakistan.

Weight was measured to the nearest 0.1kg with a digital scale with the subject wearing light clothing and without shoes. Height was recorded to the nearest 0.1cm (seca 217 stadiometer). Body Mass Index (BMI) was calculated as a ratio of weight (kg) divided by height (m^2^) and classified using the Asian criteria as proposed by Western Pacific Regional Office of World Health Organization.^7^ The waist circumference (WC) was determined while standing, at the midpoint between the top of the iliac crest and lower margin of last palpable rib, to the nearest 0.1cm. Bioelectric Impedance Analysis was done to determine body fat (%) and visceral fat (%) (Omron HBF 510 Body Composition Monitor). Normal range for body fat was taken as 10-22% for males and 20-32% for females.

Cardiorespiratory fitness was measured by submaximal exercise testing using Queen’s College Step Test, a reliable method to indirectly estimate VO_2max_. The equipment consisted of a sturdy bench 16.25 inch high, a stop watch, and a metronome set at 96 beats/min for males and 88 beats/min for females. The subjects were asked not to perform vigorous exercise twenty four hour before and not to take food, caffeine or smoke two hour before testing. Resting heart rate (HR) was noted for one minute. The subjects were then asked to step up and down in rhythm with the metronome for three minutes. Radial pulse was counted five seconds after stopping, for a period of 15 seconds and multiplied by four to get the pulse rate/min. This recovery pulse rate was used in the following equation to determine the subject’s VO_2max_ andread on an age-adjusted rating scale.^8^

In males VO_2max_ (ml/kg/min) = 111.33 – (0.42*pulse rate beats/min)

In females VO_2max_ (ml/kg/min) = 65.81 – (0.1847*pulse rate beats/min)

Statistical analyses were performed using SPSS Statistics (version 20.0). Descriptive statistics were used to evaluate the characteristics of each participant as % frequency of categorical and statistics of numeric variables. Analysis of variance (ANOVA) and Correlation Analysis was used for comparing mean and determine association between values of continuous variables across gender disaggregated VO_2max_ quartiles, with statistical significance taken at *p*<0.05.

## RESULTS

The descriptive characteristics of the study participants are given in Table-I. The mean age of participants was 19.37±0.63 years. The mean resting heart rate was 77.53±9.81 beats/minutes. The recovery heart rate at the end of exercise was significantly lower in male subjects (*p*<0.05). Accordingly, the VO_2max_ (ml/kg/min) was significantly higher in males, 55.41±9.45 as compared to females, 39.91±3.14, (p<0.001). A significantly higher proportion of females were sedentary (69%) as compared to males (33%). The overall prevalence of overweight/obesity was 44% with mean BMI of 23.16±5.08 kg/m^2^.

**Table-I:** Descriptive statistics and gender based differences in study population.

	*Males (n = 53)*	*Females (n = 80)*	*All (n = 133)*
Age, years	19.41±0.70	19.34±0.56	19.37±0.63
***Cardiorespiratory Fitness***			
Resting heart rate, beats/min	74.38±10.30	79.63±8.94	77.53±9.81
Recovery heart rate, beats/min	133.13±22.50	140.24±16.97*	137.41±19.60
Percent increase heart rate, %	79.33±21.83	77.69±25.46	78.34±24.01
VO_2max_, ml/kg/min	55.41±9.45	39.91±3.14***	46.09±9.96
***Physical Activity***			
Sedentary, n (%)	17 (32.7%)	55 (68.8%)	72 (54.1%)
Physically Active, n (%)	36 (69.3%)	25 (31.2%)	61 (45.9%)
***Anthropometric Indices***			
Weight, kg	73.01±16.04	56.47±13.34***	63.06±16.55
Height, m	172.80±7.13	158.52±5.60***	164.45±9.47
Body mass index, kg/m^2^	23.61±3.79	22.52±5.11	23.16±5.08
Body Fat, %	20.94±9.04	33.39±10.34***	28.43±11.56
Visceral Fat, %	5.98±4.01	3.64±1.65***	4.57±3.05
Waist Circumference, cm	91.46±16.01	78.41±12.27***	83.61±15.23

***Abbreviations:*** VO_2max,_ maximum oxygen uptake. Values expressed as mean±SD, unless otherwise specified. Unpaired student’s t test used to compare means between genders.

* *p*<0.05, significant; **p<0.01, very significant;

*** *p*<0.001, extremely significant

**Fig.1:**
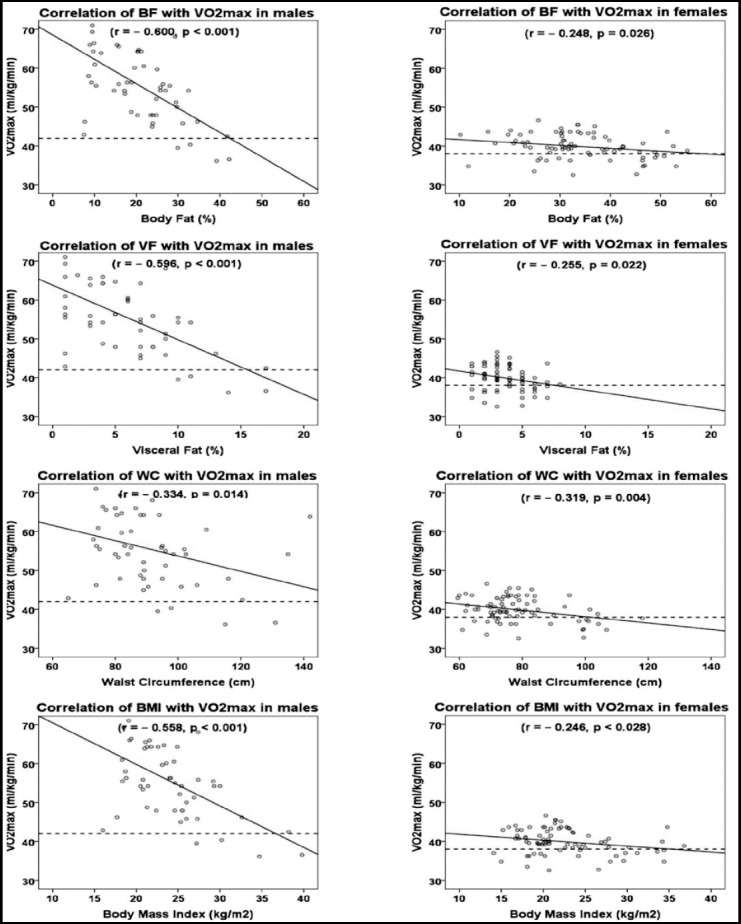
Correlation of Body Composition with VO_2max_. Scatter plot showing correlation (r) of body fat (BF), visceral fat (VF), waist circumference (WC) and body mass index (BMI) with maximum O_2_ uptake (VO_2max_) in males and females. Dotted line indicates cutoff point of VO_2max_ taken as lower limit of average VO_2max_ for this age group according to standard charts; 42 ml/kg/min in males, 38 ml/kg/min in females.

A scatterplot of Pearson’s correlation performed between adiposity indices and VO_2max_ is shown in Fig-1. A significantly negative association was seen between the two variables which was stronger in males. Of the adiposity indices the greatest correlation was seen for BF in males (*r* = – 0.600, *p*<0.001) and WC for females, (*r* = – 0.319, *p<*0.01). Lower limit of average performance was taken as the standard cutoff point of VO_2max_ for this age group; 42 ml/kg/min in males, 38 ml/kg/min in females. Only 7.5% of males had performance below average as compared to 26.3% females.

For further analysis, the subjects were divided into quartiles based of their VO_2max_ score (Table-II). There was a statistically significant difference between quartile groups for cardiac and adiposity variables as determined by one-way ANOVA. The resting heart rate, recovery heart rate and percent increase of heart rate, all decreased while going from 1^st^ quartile having lowest VO_2max_ to 4^th^ quartile with highest VO_2max_. Body composition, measured by BMI, BF, VF and WC, had an inverse relationship with VO_2max_, being high in those with lowest VO_2max_ (quartile 1) and low in those with highest VO_2max_ (quartile 4). This was further clarified by doing BMI stratification in each quartile. It was observed that in both sexes overweight and obese dominated the first two quartiles while majority of subjects in last two quartiles were of normal weight.

**Table-II:** Cardiorespiratory fitness and body composition of study participants by VO_2max_ quartile

	*1st quartile*	*2nd quartile*	*3rd quartile*	*4th quartile*	*p value*
***Males (n = 53)***					
N	13	13	13	14	
***Cardiorespiratory Fitness***					
VO_2max_, ml/kg/min	43.29±4.04	52.11±2.65	58.05±2.69	67.29±3.51	<0.001***
HRrest, beats/min	85.23±9.39	77.23±7.86	68.77±5.02	66.86±6.68	<0.001***
RHR, beats/min	162.0±9.60	141.0±6.29	126.85±6.40	104.86±8.36	<0.001***
Percent increase HR, %	91.66±18.85	84.62±23.50	85.10±12.91	57.60±13.86	<0.001***
***Adiposity Indices***					
Body fat, %	27.67±11.30	23.89±5.45	17.18±6.82	15.46±6.17	<0.001***
Visceral fat, %	9.23±5.21	6.85±2.64	4.31±2.93	3.71±2.27	<0.001***
Waist circumference, cm	97.48±17.97	95.13±15.69	90.32±19.21	83.51±6.33	0.105
Body mass index, kg/m^2^	27.92±7.15	24.73±3.06	22.45±3.50	21.57±2.43	0.002**
Underweight †	15.3%	0.0%	15.3%	0.0%	–
Normal weight †	7.6%	30.7%	38.5%	78.6%	–
Overweight †	23.0%	53.8%	30.7%	21.4%	–
Obese †	53.8%	15.3%	15.3%	0.0%	–
***Females (n = 80)***					
N	20	20	20	20	
***Cardiorespiratory Fitness***					
VO_2max_, ml/kg/min	35.84±1.56	38.98±0.56	40.87±0.85	43.94±1.00	<0.001***
HRrest, beats/min	83.05±7.89	80.25±9.96	78.00±9.34	77.20±7.89	0.160
RHR, beats/min	162.25±8.44	145.25±3.04	135.05±4.60	118.40±5.42	<0.001***
Percent increase HR, %	96.84±19.72	83.70±23.74	75.74±23.94	54.49±13.25	<0.001***
***Adiposity Indices***					
Body fat, %	36.26±13.12	35.68±8.00	32.36±9.44	29.26±9.00	0.113
Visceral fat, %	4.05±1.99	4.20±1.54	3.30±1.42	3.00±1.38	0.056
Waist circumference, cm	86.50±16.76	76.58±8.85	76.10±9.83	74.49±8.70	0.005**
Body mass index, kg/m^2^	24.28±6.58	23.19±4.45	21.81±4.58	20.80±4.12	0.145
Underweight	10.0%	5.0%	30.0%	25.0%	–
Normal weight	25.0%	50.0%	35.0%	65.0%	–
Overweight	10.0%	30.0%	25.0%	5.0%	–
Obese	40.0%	15.0%	10.0%	5.0%	–

***Abbreviations:*** HRrest, resting heart rate; RHR, recovery heart rate; VO_2max_, maximum oxygen uptake.

† Underweight (BMI<18.5kg/m^2^), normal weight (BMI=18.5-22.9kg/m^2^), overweight (BMI=23.0-27.49kg/m^2^) and obese (BMI≥27.5kg/m^2^ ). Note: Values expressed as mean±SD unless otherwise specified. One-way ANOVA between quartiles. *p<0.05, significant;

** *p*<0.01, very significant;

*** *p*<0.001, extremely significant.

## DISCUSSION

The present study was conducted to estimate the CRF in young adults. In males, the aerobic fitness was significantly higher than females (*p*<0.001). Normally, the VO_2max_ in males is 15-30% higher than in females; in our study the difference was 28% approx. This is in part due to the higher fat content in females resulting in lower lean muscle mass. Moreover, males have a greater oxygen carrying capacity due to their higher hemoglobin content. The lower performance in females seen in our study may also reflect a more sedentary lifestyle. This can be attributed to social norms and limited access to exercise facility for females.

Our findings are in agreement with other studies that have examined levels of CRF in college going young adults (Table-III). Nabi et al., (2015) assessed CRF in 18-24 year old medical students and observed VO_2max_ that was comparable to ours, especially in females. The low VO_2max_ obtained may be due to reduced physical activity and unhealthy lifestyle behaviors.^9^ Yadav et al., (2015) measured CRF in first year MBBS students. The fitness levels observed were closer to our study. However, they used Harvard Step Test protocol and the physical activity was performed for 5min.^10^

**Table-III:** Cardiorespiratory Fitness of Young Adults.

*Author/s*	*Year*	*Place of Study*	*Submaximal Exercise*	*VO_2max_ (ml/kg/min)*	

				*Males*	*Females*
Nabi T et al.^9^	2015	India	Step test	45.66±8.9	37.85±4.3
Yadav N et al.^10^	2015	India	Step test	52.11±8.80	43.70±8.28
Ko S et al.^13^	2015	Korea	Treadmill	47.7±4.9	38.4±4.3
Scott SP et al.^14^	2016	United States	Treadmill	–	44.6
Pribis P et al.^15^	2010	United States	Cycle Ergometer	38.7±11.0	34.2±10.2
Prajapati R et al.^16^		Nepal	–	54.32	44.88
Razak MRA et al.^17^	2013	Malaysia	Step test	45.4±5.1	–
This study	2016	Pakistan	Step test	55.41±9.45	39.91±3.14

***Abbreviations:*** VO_2max,_ maximum oxygen uptake. ***Note:*** Values expressed as mean±SD.

A low CRF in young adulthood is associated with an increased morbidity and mortality due to coronary artery disease later on in middle age. The Coronary Artery Risk Development in Young Adults (CARDIA) study conducted in US involved 4872 subjects aged 18-30 years. Among other variables, CRF was assessed in all and the participants were followed for approx. 29 years. It was observed that those with low CRF later on developed cardiovascular risk factors.^11^

In healthy men, adiposity and age negatively affect VO_2max_. This was demonstrated by our study where overweight and obese of both sexes had low VO_2max_ while the majority of normal weight subjects had higher fitness levels. One of the reason may be the higher lean muscle mass in subjects with lower body fat, thus increasing exercise performance. Other studies have also demonstrated an increased CRF in lean individuals as compared to their obese counterparts.^12^

Regarding body composition measured by BMI, two important observations were made: firstly, our sample consisted of 22% underweight females having a BMI<18.5 kg/m^2^. However, their exercise capacity was better than the normal weight females. Secondly, we observed that a considerable portion of students were either overweight or obese (>50% males and 35% females) having a BMI≥23 kg/m^2^ reflecting the prevalence of a major risk factor for NCDs at this young age.

While analyzing this regional data, it must also be considered that the upper limits of normal BMI are 2.0 kg/m^2^ lower for Asian population whereas the lower limits are considered the same as compared to Caucasians, i.e., 18.5 kg/m^2^. A revision may be needed in this regard to reconsider the lower limits of BMI based upon the local population as well as gender.

This study is the first to measure CRF, adiposity and gender correlations in Pakistan and hence provides a base for future research. However, our sample was limited to only one medical college. Secondly, the Queens College Step Test has its limitation of accuracy in measuring CRF although it provides the best estimate in context of simplicity and cost. Future studies on CRF involving larger number of participants of various age groups needs to be done, focusing on risk factors of cardiovascular disease.

## CONCLUSION

The present study reports a low level of overall CRF in females. Furthermore, there is a strong inverse relationship between CRF and adiposity in both genders. Since college age students are shaping their adult behaviors, the health change adopted during this period could increase CRF, decreasing the risk of chronic NCD in later years.

### Authors’ Contributions

**MRH, ZH, SZ** conceived, designed, did statistical analysis & editing of manuscript.

**MRH, ZH, SZ & MAQ** did data collection and manuscript writing, review and final approval of manuscript.
